# Mitochondrial 13513G>A Mutation With Low Mutant Load Presenting as Isolated Leber's Hereditary Optic Neuropathy Assessed by Next Generation Sequencing

**DOI:** 10.3389/fneur.2021.601307

**Published:** 2021-03-04

**Authors:** Chuan-bin Sun, Hai-xia Bai, Dan-ni Xu, Qing Xiao, Zhe Liu

**Affiliations:** ^1^Eye Center, Second Affiliated Hospital of Zhejiang University School of Medicine, Hangzhou, China; ^2^Department of Ophthalmology, Zhejiang Provincial People's Hospital, People's Hospital of Hangzhou Medical College, Hangzhou, China

**Keywords:** Leber's hereditary optic neuropathy, mitochondrial DNA, gene mutation, m13513G>A, optic atrophy

## Abstract

**Objective:** Mitochondrial 13513G>A mutation presenting as isolated Leber's hereditary optic neuropathy (LHON) without any extraocular pathology has not been reported in literature. We herein evaluate the clinical characteristics and heteroplasmy of m.13513G>A mutation manifesting as isolated LHON.

**Methods:** Seven members of a Chinese family were enrolled in this study. All subjects underwent detailed systemic and ophthalmic examinations. Mitochondrial DNA in their blood was assessed by targeted PCR amplifications, next generation sequencing (NGS), and pyrosequencing. One hundred of blood samples from ethnic-matched healthy volunteers were tested by NGS and pyrosequencing as normal controls.

**Results:** Isolated LHON without any other ocular or extraocular pathology was identified in a 16 year old patient in this family. Heteroplasmic m.13513G>A mutation was detected by NGS of the full mtDNA genome in the patient with mutant load of 33.56%, and of 26% 3 months and 3 years after the onset of LHON, respectively. No m.13513G>A mutation was detected in all his relatives by NGS. Pyrosequencing revealed the mutant load of m.13513G>A mutation of the LHON patient, his mother, father and sister were 22.4, 1.9, 0, and 0%, respectively. None of 100 healthy control subjects was detected to harbor m.13513G>A mutation either by NGS or by pyrosequencing of the full mt DNA genome.

**Conclusions:** We first report m.13513G>A mutation with low mutant load presenting as isolated LHON. NGS of the full mitochondrial DNA genome is highly recommended for LHON suspects when targeted PCR amplification for main primary point mutations of LHON was negative.

## Introduction

As one of the most common mitochondrial inherited diseases, Leber's hereditary optic neuropathy (LHON) is typically characterized by acute painless bilateral central vision loss in adolescents and young adults, predominantly in males (1–4). Previous investigations have revealed that more than 90% of LHON cases are related to one of three primary point mutations in the mitochondrial DNA (mtDNA): m.11778G>A, m.3460G>A, and m.14484T>C, which encode the ND4, ND1, and ND6 subunits of Complex I in the mitochondrial respiratory chain, respectively (5–7). However, other rare primary mtDNA mutaions such as m.3635G>A in ND1, m.14495A>G in ND6, and m.13513G>A in ND5 have also been reported to cause LHON independently (1–7).

Since first identified as a gene mutation causative for mitochondrial encephalomyopathy with lactic acidosis and stroke-like episodes (MELAS), m.13513G>A mutation has been mostly reported in Leigh syndrome (LS), MELAS, as well as MELAS/LHON and MELAS/LS overlap syndromes (5, 6). Ophthalmic manifestations related to m.13513G>A mutation including optic atrophy, ptosis, and chronic progressive external ophthalmoplegia (CPEO) are mostly accompanied by MELAS or LS, and classified as MELAS /LHON, LS/LHON, or MELAS/CPEO overlap syndromes (5–8).

Until now, m.13513G>A mutation-related LHON without LS or MELAS has been reported in only three cases (9–11). However, all above 3 cases were accompanied by other extraocular pathology such as hypertrophic myocardiopathy, myopathy, Wolff-Parkinson White syndrome, chronic kidney disease, diabetes mellitus, or hearing loss. To our knowledge, m.13513G>A mutation presenting as isolated LHON without any extraocular pathology has not been reported. We herein evaluate the clinical characteristics and heteroplasmy of the m.13513G>A mutation manifesting as isolated LHON without any other ocular or extraocular pathology.

## Methods

### Subjects and Clinical Examinations

**Figure 1:**
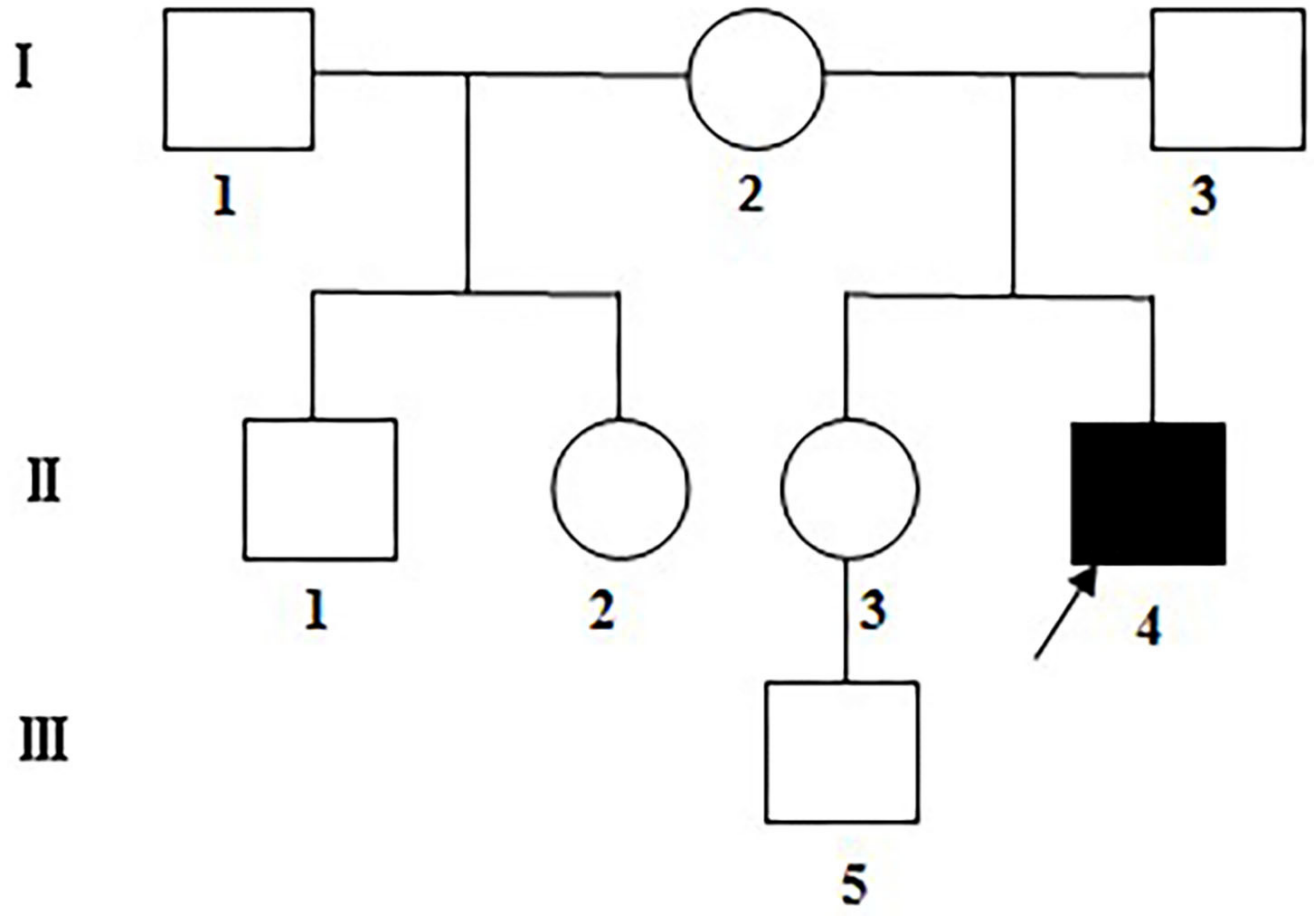
The Chinese LHON family under assessment. Visually impaired family members: filled symbols; females: circles; males: squares; arrow: the proband.

Six maternal family members (including one half-brother and one half-sister) and the farther of the LHON patient of Han population in a Chinese family were enrolled in this study (Figure 1). Detailed medical records of the patient visiting different hospitals were collected. All subjects underwent detailed systemic and ophthalmic examinations including consciousness, hearing, articulation, superficial and deep sensation, muscle strength, tone and reflexes, best corrected visual acuity (BCVA), slit-lamp microscopy, color fundus photography, and visual field test, as well as blood test for cardiac enzymes, blood lactate fluctuation during exercise test, electrocardiogram and Doppler echocardiography. Gadolinium enhanced orbital MRI, and magnetic resonance spectroscopy were performed only in the LHON patient.

### Mitochondrial DNA Assessment

Peripheral blood was collected from the patient and his relatives for mtDNA sequencing after informed consents were signed. One hundred of blood samples from ethnic-matched healthy volunteers were used as normal controls. Targeted PCR amplifications and Sanger DNA sequencing of three main primary point mutations of LHON in mt DNA, i.e.: m.11778G>A, m.3460G>A, and m.14484T>C, were first assessed in the LHON patient according to procedures described previously (12, 13). In brief, QIAamp DNA Blood Mini kits were employed for genomic DNA extraction (Qiagen, NO.51104). Three main primary mutations of LHON including m.11778G>A, m.3460G>A, and m.14484T>C were assessed by targeted PCR amplifications of DNA fragments, using oligodeoxynucleotides that correspond to mitochondrial DNA at 11,654–11,865 for m.11778G>A mutations; 3,108-3,717 for m.3460G>A mutations, and 14,260–14,510 for m.14484T>C mutations (12). Fragments were isolated and analyzed in an ABI 3700 automated DNA sequencer (Applied Biosystems; Thermo Fisher Scientific, Inc.) using Big Dye Terminator Cycle sequencing kits. Comparisons of the fragments were performed through Cambridge sequencing (Gen-Bank accession number: NC_012920).

Since none of above three primary point mutations in mt DNA was detected in the LHON patient. Next generation sequencing (NGS) of the whole mtDNA genome and DNA sequence analysis were then performed in the patient based on procedures described previously (14, 15). In brief, whole genomic DNA was extracted with the Purgene DNA isolation kit (Qiagen), and the entire genome of mitochondrial was amplified total of three pooled reactions by PCR as 99 separate fragments, using primers for the light and heavy stranded DNA. Then the DNA was purified using magnetic beads and a library was made for directly sequenced on a sequencer using a NGS reaction kit (MiniSeq, Illumina). To identify mutations within the obtained genome, the consensus Cambridge sequence (Gen-Bank accession number: NC_012920) was used as a reference (14, 15). To detect m.13513G>A mutation in the mitochondrial ND5 gene in the patient, his relatives and normal controls, 13,319–14,287 region was amplified using: forward 5′-ACA TCT GTA CCC ACG CCT TC−3, and reverse 5′- AGA GGG GTC AGG GTT GAT TC-3′, as described previously (12, 14), and analyzed as mentioned in the context.

To further detect and quantify the mutant load of the m.13513G4A mutation in the LHON patient, his relatives and normal controls, specific single nucleotide polymorphism assays using pyrosequencing which is more sensitive in detecting low-level DNA mutations were performed as described previously (16, 17). In brief, PCR amplification of a mtDNA fragment containing the m.13513 position was carried out on Pyrosequencer PSQ96MA platform using the following primers: forward 5′-CTTCAACCTCCCT CACC-3′, reverse 5′-AGCGCTGCTCCGGTTCATAGATTGCTCAGGCGTTT GTG TATGA-3′, and sequencing A(G/A)ACCACAT (nt. 13512–13520). Sequence identification was performed by the PSQ SQA software (Biotage AB), and the percentage of mutant load was determined using the quantification function of the software.

Mitochondrial DNA sequences of 17 vertebrates were assessed for interspecific analysis (13, 18). The confidence interval (CI) was measured through the comparison of human nucleotide variants to other vertebrates (*n* = 16). The CI indicates the percentage of species with wild type nucleotides at an identical position. The mitochondrial haplogroups of the Asian were also determined as described previously (19, 20).

## Results

### Clinical Characteristics of m.13513G>A Mutation-Related LHON

**Figure 2:**
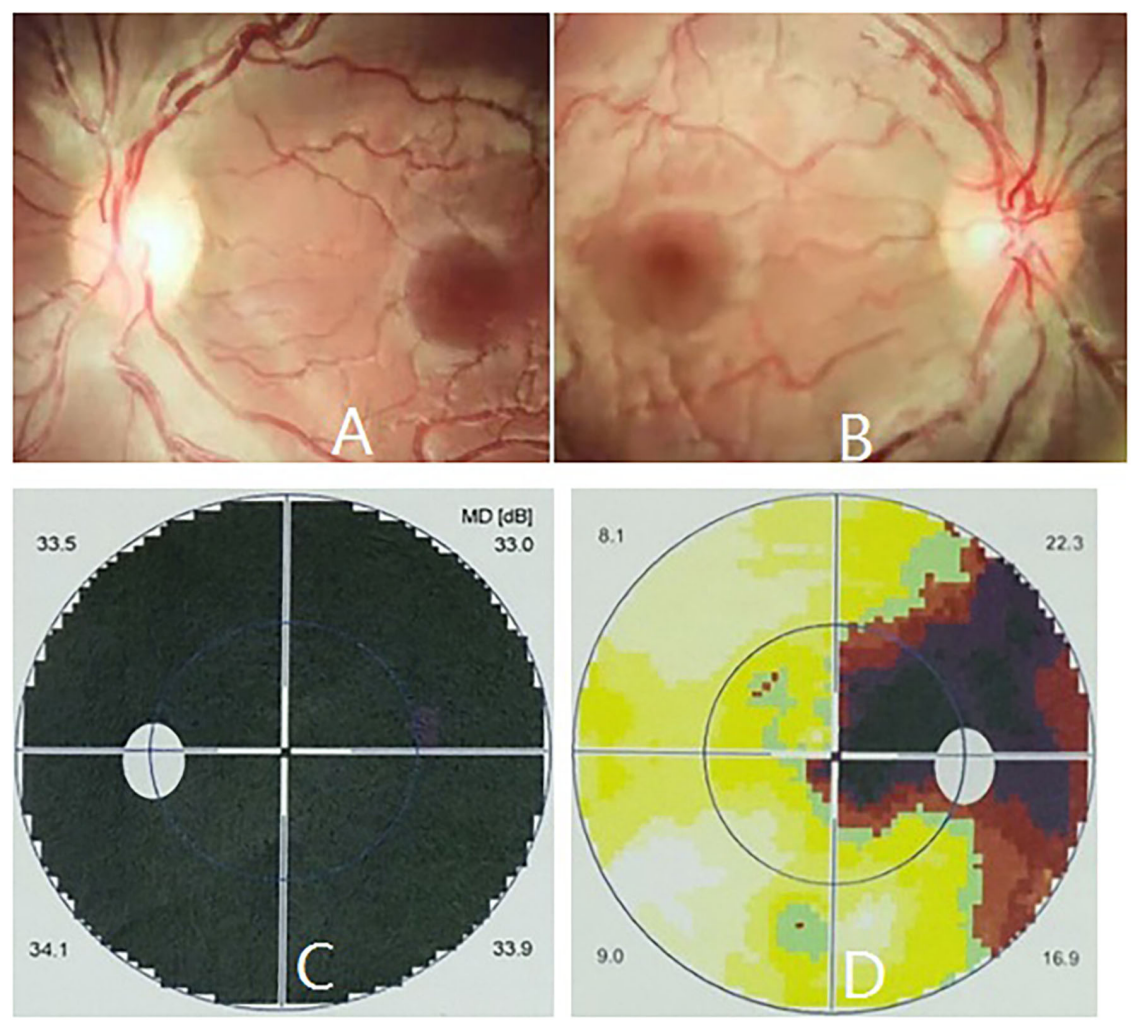
Ophthalmic manifestations of m.13513G>A mutation-related LHON at presentation. Fundus photography revealed pale temporal optic disc with congested nasal optic disc edema in the left eye **(A)**, and congested optic disc edema in the right eye **(B)**, respectively. Octopus visual field test showed full field blindness in the left eye **(C)** and large centrocecal scotoma in the right eye **(D)**, respectively.

Isolated LHON without any other ocular or extraocular pathology was identified in one patient in this family. The patient was a 16 years old male presenting with a complaint of sequential painless bilateral central vision loss. He first experienced a blurred vision in the left eye, unfortunately he did not pay any attention until another sudden vision loss in the right eye 1 month later, he was then taken to the local hospital for ophthalmic examination. Medical record revealed a BCVA of 20/100 and counting fingers in the right and left eye, respectively, fundus photography revealed congested optic disc edema in the right eye, and pale temporal optic disc with congested nasal optic disc edema in the left eye (Figures 2A,B). Octopus visual field test showed large centrocecal scotoma in the right eye and diffuse field constriction in the left eye (Figures 2C,D).

His past medical history was unremarkable, and he denied any other ocular or extraocular symptoms such as ptosis, extraocular muscle paralysis, sensation or movement disorders, epilepsy episode, or hearing loss. His physical examination was normal. Blood test for basic metabolic panel was unremarkable. Gadolinium enhanced orbital MRI examination showed normal signals of bilateral optic nerves and brain. He was initially diagnosed as bilateral idiopathic demyelinating optic neuritis, and treated with intravenous methylprednisolone pulse therapy (500 mg qd ×3d) followed by gradual tapering of oral methylprednisolone. Unfortunately, the patient showed no response to steroid therapy, and his vision progressively deteriorated in both eyes.

**Figure 3:**
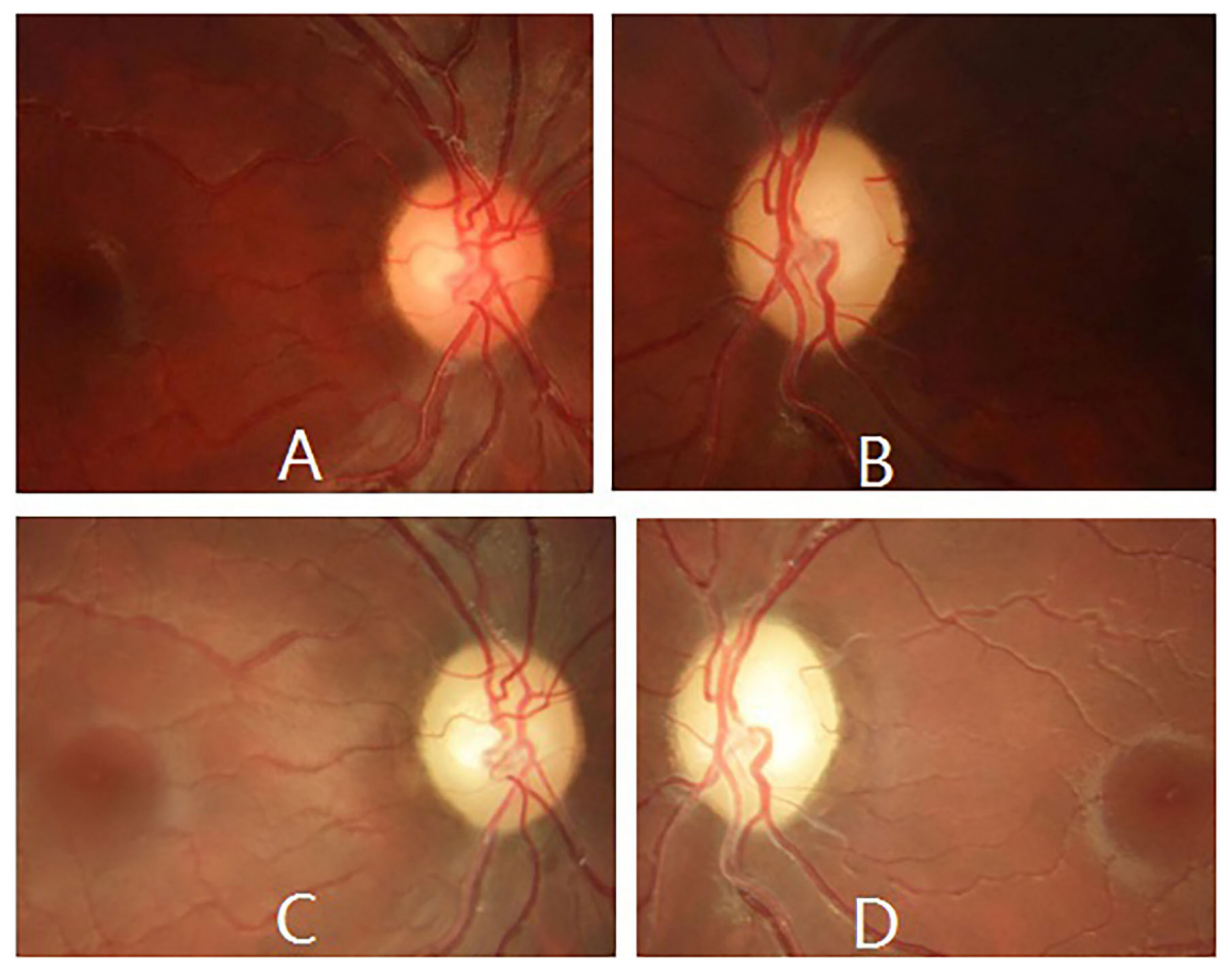
Ophthalmic manifestations of m.13513G>A mutation-related LHON during follow-up. Fundus photography revealed temporal optic disc was pale in both eyes 2 weeks later **(A,B)**, and pale optic disc in both eyes 4 years later **(C,D)**.

When the patient first came to our eye center another 2 weeks later, his vision was counting fingers in the right and hand motion in left eye, pupil size was 5 mm and pupillary light reflex was sluggish in both eyes with a relative afferent pupil defect in the left eye, and temporal optic disc was pale in both eyes (Figures 3A,B). Blood test for neuromyelitis optica-IgG antibody and myelin oligodendrocyte glycoprotein antibody were both negative. Bilateral LHON was suspected and mtDNA assessment was highly recommended. The patient initially refused our suggestion, but finally accepted and underwent mtDNA assessment 1 month later when he visited another neuro-ophthalmologist in another hospital and was clinically diagnosed as LHON.

**Figure 4:**
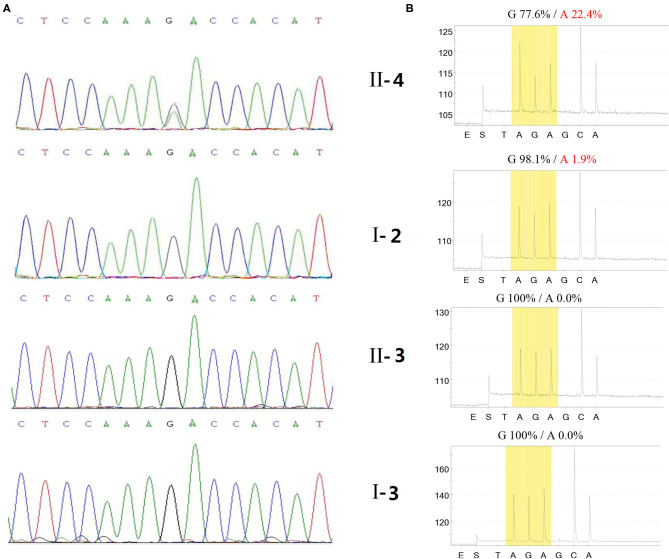
Mutant load of the m.13513G>A mutation in this Chinese LHON family. Test results of m.13513G>A mutation in the LHON patient (II-4), his mother (I-2), his sister (II-3), and his father (I-3) assessed by next generation sequencing **(A)** and by pyrosequencing **(B)**, respectively.

None of three main primary point mutations in mtDNA related to LHON was detected by targeted PCR amplifications and Sanger DNA sequencing in the patient. However, heteroplasmic m.13513G>A mutation was detected by NGS and pyrosequencing of the full mt DNA genome in this paient (Figures 4A,B). Hence, his diagnosis was corrected to m.13513G>A mutation-related LHON.

To further exclude other possible mitochondrial diseases, the patient was referred to a neurologist and a cardiologist for consultation. However, his neurological examinations were all normal including consciousness, hearing, articulation, superficial and deep sensation, as well as muscle strength, tone, and reflexes. Blood test for cardiac enzymes including creatine kinase and lactate dehydrogenase were both unremarkable. Blood lactate fluctuation was within normal limits during exercise test. Electrocardiogram was normal and Doppler echocardiography revealed normal cardiac morphology and hemodynamics. There was no lactic acid peak found in the brain by magnetic resonance spectroscopy.

The patient was regularly followed up for 4 years, ophthalmic examination at last follow-up revealed spontaneously slight increase in his vision (20/320 in the right eye and counting fingers in the left eye), and pale optic disc in both eyes (Figures 3C,D). However, no other ocular or extraocular pathology was found during his follow-up.

Although all relatives of the patient underwent detailed systemic and ophthalmic examinations, no abnormal findings in consciousness, hearing, articulation, superficial and deep sensation, muscle strength, tone, and reflexes, BCVA, anterior segment and fundus examination, visual field test, as well as blood test for cardiac enzymes, blood lactate fluctuation during exercise test, electrocardiogram and Doppler echocardiography, were found during 4 years' follow-up.

### Mitochondrial DNA Assessment of m.13513G>A Mutation-Related LHON

None of three main primary point mutations of LHON, i.e., m.11778G>A, m.3460G>A, and m.14484T>C, were detected by targeted PCR amplifications and Sanger DNA sequencing in the patient. However, heteroplasmic m.13513G>A mutation in the mitochondrial ND5 gene was detected by NGS of the full mtDNA genome and further confirmed by Sanger DNA sequencing in the patient (Figure 4A).

Mutant load of m.13513G>A mutation in the patient assessed by NGS was 33.56% (Mygenostics Company, Beijing, China) 3 months after the onset of bilateral vision loss, and 26% (Amplicon Gene Company, Shanghai, China) 3 years after the onset of bilateral vision loss. Nevertheless, no m.13513G>A mutation was detected in all his relatives by either of above twice NGS tests.

Pyrosequencing of the full mt DNA genome was assessed 3 years after the onset of bilateral vision loss in this patient, and m.13513G>A mutation with a mutant load of 22.4, 1.9, 0, and 0% was detected in the patient, his mother, father and other maternal relatives, respectively (Figure 4B). None of 100 healthy control subjects were detected to harbor m.13513G>A mutation either by NGS or by pyrosequencing of the full mt DNA genome.

**Figure 5:**
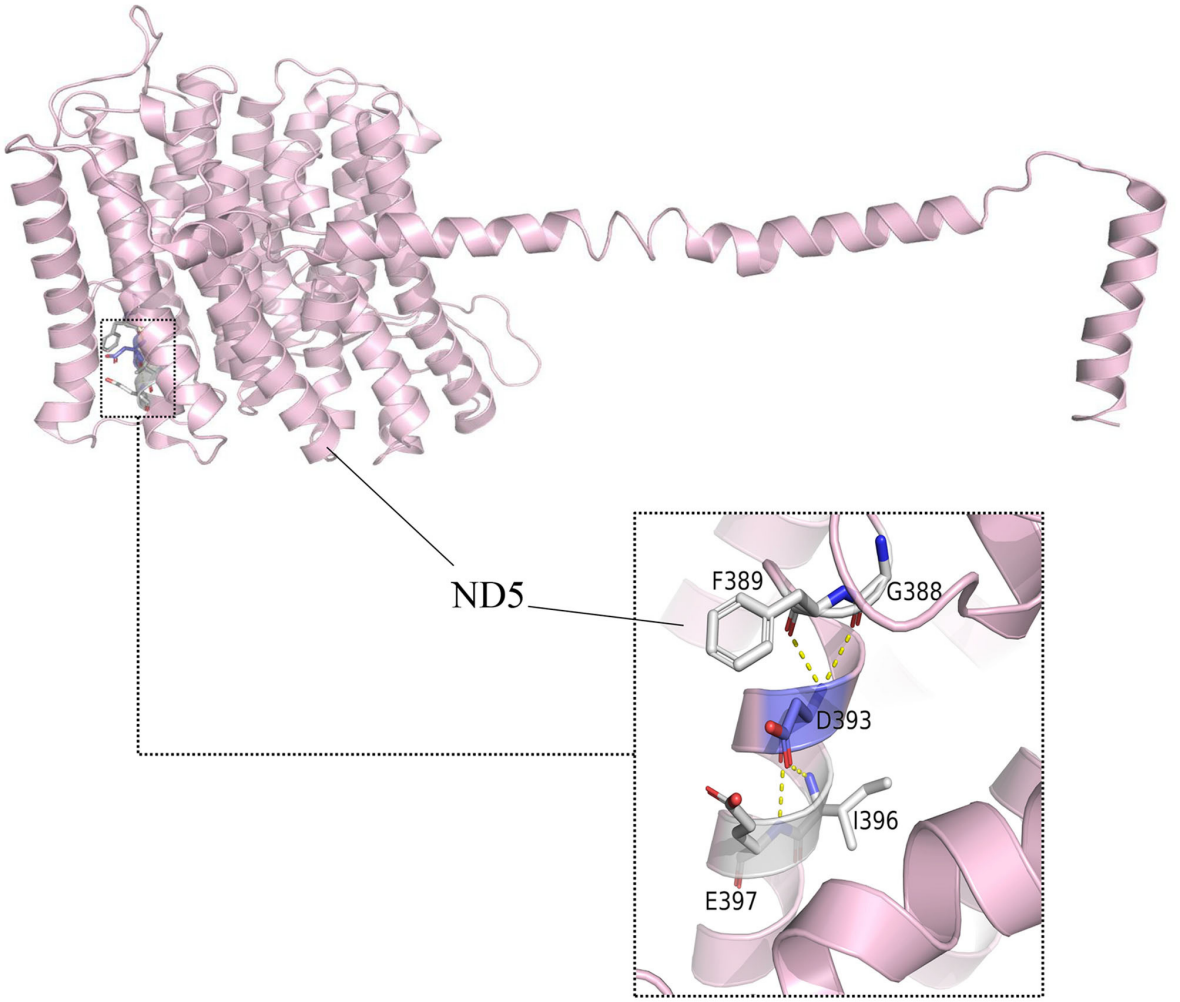
Altered complex I assembly. Structural interactions of ND5-D393 (5XTD in the protein bank). The hydroxyl group of D393 forms specific electrostatic interactions with G388, F389, I396, and E397 in MT-ND5.

Based on crystal-structures of complex I in mammals, m.13513 encoded the residue D393 of ND5 protein (Figure 5). This residue was found to be highly conserved amongst ND5 proteins of the 17 organisms. The array of polymorphic loci in the mitochondrial DNA of the family investigated in this study was shown in Table 1, there were 2 variants detected in 12S rRNA gene, 2 variants in 16S rRNA gene, 11 variants in D-loop, one variant in tRNA, and 9 missense mutations and 17 silent variants in protein- encoding genes. The phylogeny of variants were investigated through comparisons to mice (21), cows (22), and Xenopus laevis (23), whereas none of them showed obvious evolutionary conservation. Moreover, according to the nomenclature of mitochondrial haplogroups, these mtDNA polymorphic variations in this pedigree belonged to the Eastern Asian haplogroups D4a.

**Table 1:** mtDNA variants in a Chinese family with Leber's hereditary optic neuropathy.

**Gene**	**Position**	**Replacement**	**AA change**	**Conservation (H/B/M/X)**	**Previously reported**
D-loop	73	A-G			Yes
	152	T-C			Yes
	263	A-G			Yes
	298	C-T			Yes
	489	T-C			Yes
	514	C-/			Yes
	515	A-/			Yes
	16092	T-C			Yes
	16223	C-T			Yes
	16274	G-A			Yes
	16362	T-C			Yes
12S rRNA	750	A-G		A/A/A/-	Yes
	1438	A-G		C/C/A/-	Yes
16S rRNA	2706	A-G			Yes
	3010	G-A			Yes
ND1	3969	C-A			Yes
tRNA Gln	4393	C-T			Yes
ND2	4769	A-G			Yes
	4883	C-T			Yes
	5178	C-A	Leu-Met	L/T/T/T	Yes
	5231	G-A			Yes
CO1	7028	C-T			Yes
ATP8	8414	C-T	Leu-Phe		Yes
ATP6	8701	A-G	Thr-Ala	T/S/L/Q	Yes
	8860	A-G	Thr-Ala	T/A/A/T	Yes
CO3	9355	A-G	Asn-Ser		Yes
	9380	G-A			Yes
	9540	T-C			Yes
ND3	10398	A-G	Thr-Ala	T/T/T/A	Yes
	10400	C-T			Yes
ND4	10873	T-C			Yes
	11059	C-T			Yes
	11719	G-A			Yes
ND5	12705	C-T			Yes
	13104	A-G			Yes
	13513	G-A	Asp-Asn		Yes
	13708	G-A	Ala-Thr		Yes
ND6	14668	C-T			Yes
Cytb	14766	C-T	Thr-Ile	T/S/T/S	Yes
	14783	T-C			Yes
	15043	G-A			Yes
	15301	G-A			Yes
	15326	A-G	Thr-Ala	T/M/I/I	Yes

## Discussion

Although ~90% of the mtDNA mutations related to LHON was one of three primary mutations, i.e., m.11778G>A, m.3460G>A, and m.14484 T>C, other rare primary mtDNA mutaions including m.13513G>A have also been reported to cause LHON independently (1–7). As the most frequently reported mutation in the mitochondrial ND5 gene, m.13513G>A mutation was identified as a causative gene mutation mostly related to LS, MELAS, and LS or MELAS related overlap syndromes (7–11, 24, 25). Until now, LHON caused by m.13513G>A mutation yet not accompanied by LS or MELAS was reported in only three cases (9–11). However, all above three LHON cases were accompanied by other ocular and (or) extraocular pathology (9–11). To our knowledge, this is the first report of the m.13513G>A mutation presenting as isolated LHON yet without any other ocular or extraocular pathology.

In mitochondrial diseases, it is not unusual for the same mutation in the same gene to result in different manifestations. Previous study indicated that germ-line mtDNA bottleneck existed during oogenesis and caused significant heteroplasmy frequency shifts between generations. As for a pathogenic heteroplasmy, a severe bottleneck might abruptly transform a benign (low) frequency in a mother into a disease-causing (high) frequency in her children (26). It is also hypothesized that the drastic changes of heteroplasmy frequency between generations contribute to non-disease mutant load or higher disease severity. In this study, the LHON patient presented 22.4% mutant load, compared to his mother only 1.9% in blood cells by pyrosequencing assessment, which might be explained by the genetic drift due to germ-line bottleneck effect.

The m.13513G>A is a point mutation in mitochondrial ND5 gene which can cause severe oxidative phosphorylation defect (26). The amino acid at position 393 of ND5 subunit encoded by m.13513 is located at an evolutionarily conserved part of a putative quinone-reactive site of the enzyme, and the D393N amino acid change caused by m.13513 G>A mutation may lead to loss of the quinine reactive site and a subsequent negative effect on the activity of the oxidative phosphorylation system, followed by significant mitochondrial impairment, as well as an increased reactive oxygen species generation and reduced ATP production, which finally resulting in the development of LHON (24, 25, 27).

In cultured cells, the threshold for m.13513G>A mutation causing a complex I defect is a mutant load of ~30%, and its impairment increased in a mutant-load dependent way (24, 28). The mutant load of the m.13513G>A mutation which is 22.4 ~33.56% in blood cells in the LHON patient in our study is consistent with above finding. Previous investigations about the mutant load in patients with m.13513G>A mutation revealed much lower mutant load of 4~6% in blood cells, 1~5% in fibroblasts, and 13~15% in muscle in one patient, and of 11~17% in blood, hair and muscle tissues in another patient (24). Among all human tissues, optic nerve head is most susceptible to mitochondrial dysfunction, since mitochondria are most abundantly clustered in retinal nerve fibers at optic nerve head. On the other hand, previous investigations have demonstrated that ND5 synthesis is probably the rate-limiting step for the activity of complex I and consequently of respiration (24). Hence, contrary to most pathogenic mtDNA mutations which only result in the outbreak of a oxidative phosphorylation defect disease when presenting at high mutant loads in target tissues, the m.13513G>A mutation may cause significant complex I defect at optic nerve head and induce an isolated LHON without any other tissue involvement even at unusually low mutant loads.

Our study revealed that the mitochondrial genomes of this pedigree belongs to the Eastern Asian haplogroups D4a. Although many other mtDNA variants were also detected in this study, there were no functionally significant mutations in this pedigree. Hence, these mtDNA variants may not have a potential modifying role in the development of visual impairment associated with m.13513G>A mutation. This implied that m.13513G>A mutation, similar to three primary mt.DNA mutations related to LHON, occurred sporadically and multiplied through evolution of the mtDNA.

There are several limitations in this study. First, only peripheral blood cells were collected for genome assessment. Considering that the percentage of m.13513G>A mutation may vary in different tissues, it would greatly benefit if the percentage of heteroplasmy of m.13513G>A mutation in other tissues such as urine, hair, buccal mucosa, and muscles were assessed. Unfortunately, the patient declined further genome assessment, for no other tissues except optic nerves were involved. Second, although no other ocular or extraocular pathology was found in this young-adult patient during 4 years' follow-up, other tissues involvement can not be excluded if follow-up time is much longer. Hence, regular ophthalmic and systemic examinations, as well as long-term follow-up have been highly recommended to this patient.

In summary, low mutant load of m.13513G>A mutation was detected in the LHON patient by both NGS and pyrosequencing of the full mt DNA genome in this study, indicating that NGS and pyrosequencing are both sensitive to detect low-level DNA mutation. Hence, patients with typical ophthalmic manifestations of LHON but with negative results of three primary point mutations related to LHON tested by targeted PCR amplification, may need to be further assessed by NGS or pyrosequencing.

## Data Availability Statement

The raw data supporting the conclusions of this article will be made available by the authors, without undue reservation.

## Ethics Statement

The studies involving human participants were reviewed and approved by Second Affiliated Hospital of Zhejiang University School of Medicine. Written informed consent to participate in this study was provided by the participants' legal guardian/next of kin.

## Author Contributions

C-bS and ZL wrote and reviewed the manuscript. C-bS, H-xB, and D-nX collected the patient data. C-bS, D-nX, and QX performed literature research. C-bS, H-xB, and ZL analyzed and interpreted the clinical data. C-bS performed medical treatment. All authors read and approved the final manuscript.

## Conflict of Interest

The authors declare that the research was conducted in the absence of any commercial or financial relationships that could be construed as a potential conflict of interest.
